# Using Generative Modeling to Endow with Potency Initially Inert Compounds with Good Bioavailability and Low Toxicity

**DOI:** 10.1021/acs.jcim.3c01777

**Published:** 2024-01-23

**Authors:** Robert I. Horne, Jared Wilson-Godber, Alicia González Díaz, Z. Faidon Brotzakis, Srijit Seal, Rebecca C. Gregory, Andrea Possenti, Sean Chia, Michele Vendruscolo

**Affiliations:** † Centre for Misfolding Diseases, Department of Chemistry, University of Cambridge, Cambridge CB2 1EW, United Kingdom; ‡ Imaging Platform, Broad Institute of MIT and Harvard, Cambridge, Massachusetts 02142, United States; § Bioprocessing Technology Institute, Agency for Science, Technology and Research (A*STAR), 138668 Singapore, Singapore

## Abstract

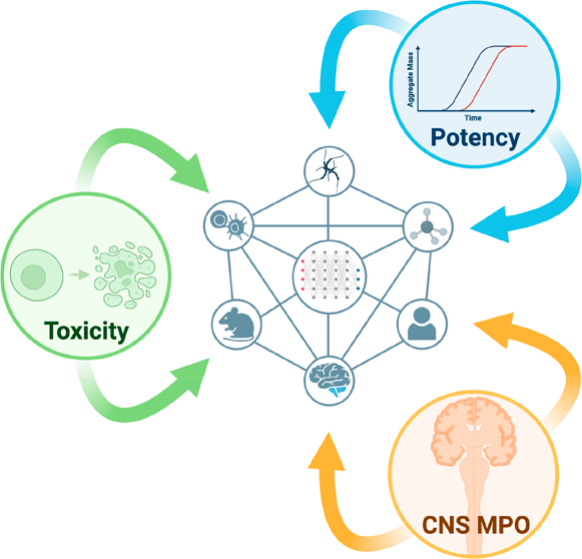

In the early stages of drug development, large chemical libraries are typically screened to identify compounds of promising potency against the chosen targets. Often, however, the resulting hit compounds tend to have poor drug metabolism and pharmacokinetics (DMPK), with negative developability features that may be difficult to eliminate. Therefore, starting the drug discovery process with a “null library”, compounds that have highly desirable DMPK properties but no potency against the chosen targets, could be advantageous. Here, we explore the opportunities offered by machine learning to realize this strategy in the case of the inhibition of α-synuclein aggregation, a process associated with Parkinson’s disease. We apply MolDQN, a generative machine learning method, to build an inhibitory activity against α-synuclein aggregation into an initial inactive compound with good DMPK properties. Our results illustrate how generative modeling can be used to endow initially inert compounds with desirable developability properties.

## Introduction

High-throughput screens are often the beginning of drug discovery pipelines, marking the division between the exploratory research and drug development stages.^1,2^ These screens often yield hit compounds that are not yet drug-like, leading to a laborious process of optimizing pharmacokinetics, pharmacodynamics, and toxicology, frequently with a significant concomitant loss of potency.^3^ Attempts can be made to optimize drug potency simultaneously with metabolism and pharmacokinetics (DMPK), but this creates challenging situations where different optimization metrics are often in opposition.^4^

As a result, for some areas of drug development it may be of interest to take a more conservative approach, starting from regions of the chemical space that already possess a strong DMPK profile and screening for potency in proximal areas of the chemical space. This approach may be particularly helpful in therapeutic areas that require target engagement in the central nervous system (CNS), as the modifications required for CNS accessibility may ablate a significant proportion of the potency that time and resources had been invested in obtaining.^5^ CNS-accessing compounds are of special interest for brain disorders, including neurodegenerative diseases, chronic pain, depression, and schizophrenia.^6−8^ In most cases, there remains an unmet need for treatments in these areas, in part resulting from challenges in understanding biological mechanisms of disease, but also due to the blood brain barrier which blocks access to most of small molecule drugs, and nearly all of macromolecule therapeutics.^9,10^ In such a scenario, it would be interesting to start from strong DMPK properties before investigating potency during drug development, to help ensure that drugs reaching the end of the pipeline engage their targets effectively and in order to reduce the requirement for invasive delivery strategies.^9,10^

In this work, we aim to provide an example of a generic “null library” that contains inert (null) molecules with good bioavailability. We define as inert compounds having minimal biological side effects, i.e., not likely to hit any off targets that would hamper clinical trials such as G protein-coupled receptors (GPCRs), kinases, ion channels, and transporters, which are critical to cell function.^11,12^ Given the sensitivity of the CNS to toxicity it is important that these off targets are minimised.

Three methods could be pursued here to fulfill these criteria: (1) parsing of clinical trial data to identify compounds with good safety profiles, (2) using machine learning models to carry out in silico screening of libraries and predict compounds with desired properties such as low toxicity,^13,14^ and (3) using generative modeling to create molecular structures with predicted potency beginning from structures with strong DMPK properties.

In this work, we explore these three approaches in the case of α-synuclein (αS) aggregation, a process implicated in Parkinson’s disease. Misfolded oligomeric aggregates of αS disrupt membranes within neurons, especially those of mitochondria,^15,16^ while the highly ordered fibrillar aggregates act as catalytic surfaces for the production of further oligomeric aggregates.^17^ αS is a challenging target, with successive hurdles of the blood brain barrier and the neuronal membrane to overcome. The training data for potency was a small set of aggregation inhibitor data generated previously.^18−21^ The assay used to generate this data set was also used in this work to test compounds predicted to be potent. For DMPK properties, we used a computational toxicity filter, the central nervous system multiparameter optimization (CNS MPO) score,^22^ and an experimental metabolic assay tracking ATP levels in cells as a proxy for cell viability (see Materials and Methods).

Based on our results, we note that the first process tends to be laborious and necessarily limited in terms of molecular diversity sampled. Final stage drug candidates are also rarely good starting points for elaboration, given their pre-existing complexity. This approach would primarily be suitable for repurposing efforts. For exploring the second approach we used biological data in combination with chemical structures.^47^ We started from previous work^14^ that employs random forest models trained on a combination of Morgan fingerprints,^23^ Cell Painting,^24^ and gene ontology features to classify molecules as toxic or nontoxic. Although similar approaches for combining data have been shown to improve accuracy on a range of bioactivity predictions,^48^ this approach needs to be established individually for each bioactivity endpoint studied. When using this strategy to find compounds targeting αS, we failed to identify compounds that were both efficacious and nontoxic. By contrast, we found that the third approach, implemented in terms of structural alterations via generative models such as MolDQN^25^ or MolCycleGAN,^26^ could be rather promising.

## Results

### Identification of Starting Points for Repurposing from a Set of Clinical Molecules

As a demonstration of the first approach and its limitations, an initial library of compounds fitting the null library criteria of good safety profile and few gene targets was identified by mining the repositioning database collated by Brown and Patel^27^ and the Drug Repurposing Hub^28^ (Figure S1). The repositioning database was filtered to obtain drugs that failed in phase I, II, or III without safety concerns, forming a library of ∼500 molecules, which was further curated to remove toxic cancer treatments and all biologics. Of the curated compounds from the repositioning library, there was ∼80% overlap with the Drug Repurposing Hub. However, 57% of these compounds caused changes in expression of more than one gene, and so they were removed as they could not be considered as inert. Molecules reaching Phase II or III in the Drug Repurposing Hub with changes in expression of up to one gene were also included, giving a final library size of ∼600 for potential use in repurposing or limited elaboration projects. We decided to not pursue this strategy further, since this subset was limited in terms of both data set size and ease of functionalization against a desired target, as well incomplete data on changes in gene expression and opaque reporting on clinical trial failure.

### In Silico Screening for Molecules with Low toxicity

The second approach that we attempted was to filter compounds from the Cell Painting (CP) data set based on their predicted toxicity using an approach recently developed.^14^ As a benchmark, we then directly selected for compounds that passed the toxicity filters and also showed aggregation inhibition potential using a QSAR model^21^ trained on the aggregation inhibitor data set. We found four of the compounds predicted to have low toxicity also to have good predicted potency.

The structures within the CP data set deviated significantly from those in the aggregation data set, implying that the generalizability of the model to this search space would be poor. One compound (ISF1, Figure S2A), from among this number appeared to exhibit aggregation inhibition (Figure S2B, C), which is shown in comparison to the positive control compound Anle-138b,^29^ an αS aggregation inhibitor in clinical trials. However, the scaffold of ISF1 is notoriously cytotoxic. This issue was not identified by the model trained on the CP data set, suggesting limitations in the model or in the ability of the CP features to encode information about long-term toxicity. Possible limitations in the CP features may arise from coarse granularity of the readouts, so that more subtle toxicity mechanisms are missed or because the data are limited to a single cell line, thus not encompassing possible toxic effects in different cell types.

A further crucial filter for compounds designed to target αS aggregates in neurons is bioavailability. In this case we use a measure of brain blood barrier permeability, implemented here via a CNS MPO score.^22^ ISF1 had a poor CNS MPO score of 1.8, largely due to its high molecular weight, high topological polar surface area (TPSA, a measure of the polar surface of a compound), and high logP (a measure of the lipophilicity of a compound, expressed as the logarithm of its partition coefficient between n-octanol and water). The common CNS MPO score cut off in terms of a viable CNS penetrant compound is 4 out of a possible total of 6,^22^ based on the sum of six molecular parameters scored between 0 and 1. This demonstrated the challenges of a direct search for a molecule with high potency and good DMPK, which failed to produce any leads.

### Potency Optimization of an Initial Inert Small Molecule Using MolDQN

Given the drawbacks of the previous two strategies, we sought to combine experimental and computational methods to validate the null criteria for a compound: bioavailability and low toxicity. To this end, an initial parent structure with no experimental toxicity, strong CNS MPO score, and no predicted or experimental activity against aggregation was chosen to observe whether it could be functionalized to some degree against the chosen target. We used a generative modeling method (MolDQN, see Materials and Methods) to move from the parent chemical space with strong DMPK toward higher potency, yielding a weighted compromise between the two via multiparameter optimization of CNS MPO score and potency. MolDQN uses deep Q learning, where each compound encountered by the model is a state and every possible modification to the compound constitutes the set of possible actions. It generates a set of compounds derived from a starting structure which have the desired properties, as predicted by a set of QSAR models; feed forward neural networks with ReLU activation were used for this task, employing mol2vec structural embeddings (see Materials and Methods). The train–test scores for the metrics of interest are shown for different optimized benchmarking models in Table 1 and Figure S3, with hyperparameters for the neural networks shown in Table 2. MolDQN was chosen due to the ease with which changes could be made to the reward function, allowing simultaneous optimization of the priority metrics such as potency and CNS MPO, but also synthetic accessibility (Figure S4) and predicted binding to the target, provided by AutoDock Vina.^30^

**Table 1:** Training and Testing Scores for QSAR Models Tested on Different Parameters of Interest^a^

	Data set MSE					
Model	AutoDock Vina train	AutoDock Vina test	CNS MPO train	CNS MPO test	Aggregation train	Aggregation test
LR	0.699	0.878	0.366	0.416	0.0002	O(10^11^)
DT	0.009	0.359	0	0.487	0.0002	0.122
RF	0.047	0.178	0.28	0.232	0.052	0.516
DNN	0.304	0.415	0.056	0.123	0.017	0.081

a Average mean square error (MSE) from cross validation for four different models for the AutoDock Vina scores and CNS MPO scores (training and testing on the Cayman dataset of ∼10,000 compounds) and half-times of aggregation (training and testing on the aggregation dataset of ∼300 compounds^18−21^). LR = linear regressor, DT = decision tree, RF = random forest, and DNN = deep neural network.

**Table 2:** Optimized Hyperparameters for Training of QSAR Neural Networks^a^

	QSAR model parameters			
Data set	Layers	Nodes per layer	Activation	Learning rate
AutoDock Vina	3	256, 256, 32	ReLU, Sigmoid	1 × 10^–3^
CNS MPO	3	256, 256, 32	ReLU6	1 × 10^–3^
Aggregation	4	128, 128, 128, 32	ReLU, Sigmoid	5 × 10^–4^

a Models were trained for 1000 epochs.

AutoDock Vina gives a predicted binding energy to a target pocket, in this case a common binding pocket (Figure S5) identified in two amyloid fibril structures of αS obtained by cryo-electron microscopy - 6cu7^31^ and 8a9l.^32^ The latter was found to be prevalent in diseased brains containing Lewy bodies.^32^ Both amyloid polymorphs are able to accelerate aggregation by offering a surface for formation of further aggregates, in a process called secondary nucleation.^17^ Compounds targeted at this common site were previously found to inhibit aggregation catalyzed by both fibril types,^21^ in accordance with the hypothesis that these compounds are inhibitors of secondary nucleation.^18^ The set of obtained compounds are enriched in inhibitors, giving a hit rate of ∼5%^18^ compared to high-throughput screening (HTS) hit rates of <0.5% for this target.^33^ The AutoDock Vina binding energy was therefore included to give a larger data set to train on with relevance to the task at hand, which is the identification of compounds with increased likelihood of binding to fibrils and preventing secondary nucleation. In this case, the binding scores were calculated for the drug-like Cayman^34^ data set (8231 compounds). This metric was optimized alongside CNS MPO scores also derived for the Cayman data set, and the experimental potency metric, the normalized half-time of aggregation (*t*_1/2_), from a separate set of 225 inhibitors.^18−21^ The normalized half time is the time taken for half of the monomer to convert to fibril in the presence of the compound divided by the same time point for the negative control.

Summary results for the MolDQN output are shown in Figure S6, with the original data distributions of the Cayman set training population (blue) and the QSAR model predicted distributions on the generated population (orange) for the different parameters of interest that were being simultaneously optimized. An example subset of the generated compounds is shown in Figure S7, with a schematic for the pipeline starting from the inert parent compound with a perfect CNS MPO score and low predicted inhibition and experimentally validated low toxicity. We began with the Cayman set to obtain a parent structure, and derivatized it using MolDQN, as this set is considered more drug-like and so more likely to fulfill the overriding goal of this project of developing compounds with good bioavailability and fewer critical off targets. However, while the generated structures were synthetically accessible, they were not commercially available. The Cayman set is limited in its diversity, size and availability so any exploration within it would also not be expected to yield results. To address this issue, we ran a similarity search of the generated compounds using Tanimoto similarity (ECFP4 fingerprints, bits = 2048, radius = 2) on the ZINC15^35^ database, to identify similar structures that were purchasable. This set is considerably larger and more diverse and has greater availability. The most similar structures within ZINC to the parent and generated compounds, shown in Figure S8, are within a Tanimoto similarity threshold of 0.40. Previous studies indicated that a cutoff of Tanimoto similarity ≥0.40 removes compounds significantly dissimilar.^36,37^ Another study indicated that a Tanimoto similarity threshold of 0.43 (when calculated using ECFP4) was sufficient to detect half of the maximal active pairs in an internal library of over 150,000 compounds and 23 protein targets.^38^ The compounds in the resulting data set were then further filtered using mol2vec structural representations and a previously developed QSAR model,^21^ fitted to the original aggregation data. Seven of the compounds predicted to be potent were obtained. We note that using a relatively low threshold for the Tanimoto similarity with ECFP4 fingerprints, as we did here, could select compounds with rather different bioavailability and toxicity properties from the initial ones but also lowers the chances for false positives.^39^ One could circumvent this problem by having custom-made the compounds generated by MolDQN.

**Figure 1:**
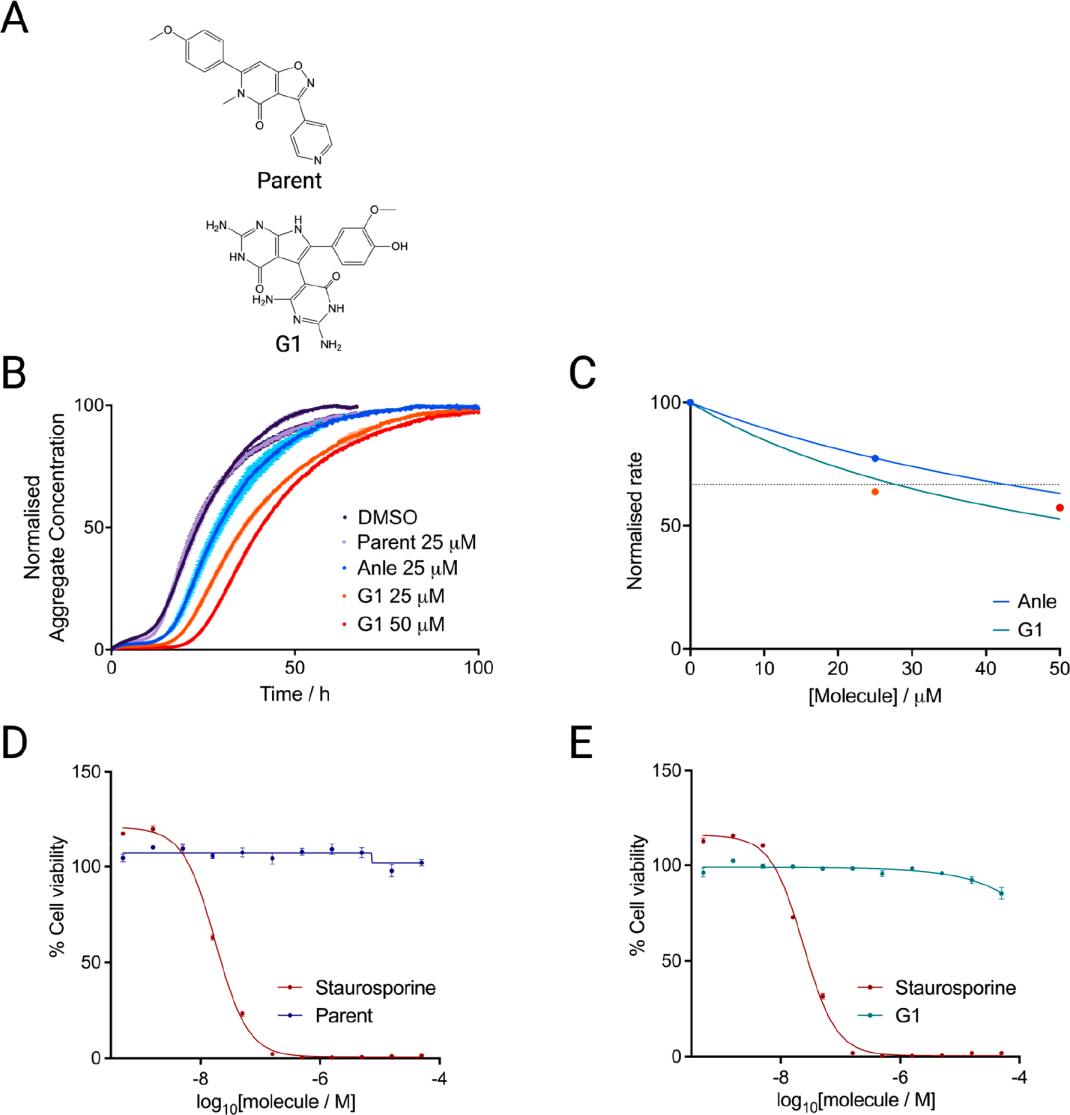
Characterization of the most potent compound identified by the generative elaboration of an inert compound. (A) Structures of the parent compound and its derivative hit (G1). (B) Kinetic traces of a 10 μM solution of αS with 25 nM seeds at pH 4.8, 37 °C in the presence of G1 at the concentration indicated (orange, red) or 1% DMSO (purple). Anle-138b (blue) is shown as a positive control and the inert parent compound is shown for comparison (lilac). (C) Approximate rate of reaction (taken as 1/*t*_1/2_, normalized between 0 and 100) in the presence of Anle-138b (blue) and G1 (teal). The data point colors match those in panel B. The KIC_50_ of G1 (27.9 μM) is indicated by the intersection of the fit and the horizontal dotted line. Anle-138b has an extrapolated KIC_50_ of 42.86 μM based on the sample tested here. (D) Human neuroblastoma cells (SH-SY5Y) at a final density of 20,000 cells/well and incubated in the presence of the generative modeling parent compound (navy) for 24 h, before addition of CellTiterGlo to detect ATP levels as a proxy for cell viability (see Materials and Methods). The concentration range is shown as a log scale, from 500 pM to 50 μM. Staurosporine, which induces apoptosis, is shown as a negative control (maroon). (E) Cell viability in SH-SY5Y cells after 24 h of incubation with G1 (teal).

The results described below show that it was possible to derive a compound with good potency from this inert starting compound with good DMPK properties, including low toxicity and good CNS MPO score. This derived compound, G1 (Figure 1A), had intermediate CNS MPO score (3.29), and improved potency compared to Anle-138b in this experiment (Figure 1B). Aggregation kinetics are shown in Figure 1B, while Figure 1C shows an approximate overall rate of aggregation at different concentrations of Anle-138b and G1. This approximate rate was taken as 1/*t*_1/2_, and fitted to a Hill slope. A kinetic inhibitory constant (KIC_50_) - the concentration of compound at which the *t*_1/2_ is increased by 50% with respect to the negative control as defined previously^40^ - was then derived.

To ascertain cell viability upon treatment with these compounds, human neuroblastoma cells (SH-SY5Y) at a cell density of 20k/well were incubated for 24 h with the inert parent compound used at the start of generative modeling (Figure 1D) and its derivative, G1 (Figure 1E). Staurosporine, which induces apoptosis, was used as a negative control. Both the parent sample and the G1 sample exhibited low toxicity, with no reduction in viability up to 50 μM for the parent, and a small reduction in viability for G1 observed at the higher end of the range tested, falling below 90% only at 50 μM. G1 therefore retained the low toxicity of its parent while gaining functionality against the target. Additional experiments at lower cell density are shown in Figure S9, with similar outcomes.

We found that four of the seven predicted hit compounds tested exhibited inhibition (Figure 1B and Figure S10), with a potency approaching Anle-138b or better in the case of G1. The core of the structures resembled those generated by MolDQN, with the transfer of one aromatic ring group to the other side of the structure and alterations of the heteroatom number and distribution. While these compounds did exhibit aggregation inhibition and low toxicity, these structural changes also led to a drop in CNS MPO scores. For example, the aggregation inhibitor G1 had a CNS MPO score of 3.29, compared to the perfect score of 6 of its parent. The issue with the structure of this compound was the TPSA and the number of hydrogen bond donors, both of which could be addressed by reducing the number of NH groups present within the aromatic ring systems. These issues would have to be addressed by custom synthesis, which would remove the need for the intermediate step to map the generated compounds onto what was commercially available and rescreen them through QSAR models. However, this was outside the scope of this work, which was intended to illustrate a proof of concept of pushing an inert compound with good DMPK properties toward target activity.

## Discussion and Conclusions

We have presented an approach to start a drug discovery program from a compound with strong DMPK properties, as a means of derisking a pipeline.

Our initial attempt consisted of a repurposing strategy for drugs with poor efficacy against their original targets. This attempt was found to be problematic due to reliance on manually parsed, poorly recorded clinical trial data with limited data set size. Furthermore, the complexity of endpoint drugs did not predispose them to be favorable candidates for structural alterations.

Our second attempt was aimed at improving the diversity of compounds and the size of the data sets available as, at least in principle, any compound data set could be screened using a toxicity predictor, provided the compounds within that data set had similar substructures to what the predictor was trained on. After screening through a toxicity predictor trained on Cell Painting cell perturbations, the predicted nontoxic fraction was then screened through a QSAR model trained on the potency metric of interest. However, out of the compounds that could be obtained, the only compound that exhibited activity had a poor CNS MPO score and high cytotoxicity concerns. These results illustrate the challenge of attempting a direct search for a compound with ideal properties by using computational screening alone.

Based on the lessons from the first two attempts, in our third attempt, we used a generative model to push a population of compounds or a single structure from a position of strong DMPK and low toxicity toward a position of desired potency. This approach yielded a better compromise between DMPK, toxicity, and potency. We used a single structure as a starting point for ease of experimental illustration, but this could equally be done with a population of compounds with desired properties using newer models such as a more recently reported chemical language generative model.^41^ As a result of the need to find purchasable material via the similarity screen and subsequent QSAR filtering, there were structural deviations from the original generated structures, including the relocation of one of the aromatic groups and changes in the heteroatom distribution. There were also difficulties retaining a high CNS MPO score at this stage. Indeed, while the MolDQN implementation made conservative changes to a core structure, those changes tended to involve addition of polar groups to mimic the properties of the aggregation inhibitor set, which had higher polar surface area in general. These changes had a harmful effect on the CNS MPO if employed excessively. To more appropriately pursue the strategy outlined in this work, a more stringent weighting would be applied to the CNS MPO to ensure this was degraded as little as possible during potency optimization, and the structures themselves would then be synthesized rather than utilizing similarity searches to find the closest option.

Overall, the aim of this work was to demonstrate that, starting from compounds with strong DMPK properties, it is possible to move toward compounds of promising potency. This approach could be seen as the reverse of more commonly used approaches, which start from compounds with promising potency and then optimize their DMPK properties. In both scenarios, machine learning can be a great aid in identifying promising chemical matter.

We have demonstrated this approach by modifying a compound with strong CNS MPO score and low experimental toxicity from the Cayman set of drug-like compounds to obtain potency in an assay relevant to drug discovery for Parkinson’s disease. We anticipate that future approaches could utilize generative adversarial networks to bias inert compound populations toward regions of the chemical space with higher potency while controlling the distance from the desirable DMPK space.

## Materials and Methods

### Prediction Models

All coding was carried out in Python 3. Neural networks were created with Pytorch. Scikit-learn^1^ implementations of random forest, decision tree, and linear regressors were tested for benchmarking and filtering of molecules after the similarity searches (see Supporting Information). For data handling, calculations, and graph visualization the following software and packages were used: pandas,^42^ Seaborn,^43^ Matplotlib,^44^ NumPy,^45^ SciPy,^46^ and GraphPad Prism 9.1.2.

### MolDQN

MolDQN was not altered from the published version aside from the tailoring of parameters and parameter weights of the QSAR models to optimize the metrics of the generated compounds such as the aggregation half time, CNS MPO score, binding score, and synthesisability score.

Experimental methods can be found in the Supporting Information.

## Data Availability

Code and data for the toxicity filtering can be found at https://git.io/Jkra8. Code and data for subsequent generative modeling can be found at https://github.com/Jaredwg2000/MolDQN_CNS. Code and data for the previously developed QSAR filter can be found at https://github.com/rohorne07/Iterate.

## Supporting Information Available

Metrics for molecules collated during manual clinical trial parsing, model performance metrics on the different data sets, aggregation data for ISF1 effects of introduction of a synthetic accessibility penalty, structural representations of the fibril binding pockets, SH-SY5Y toxicity data at different cell densities, metrics for generated molecules, a summary pipeline schematic for the third outlined approach, and aggregation data for the milder inhibitors identified via generative modeling (PDF)

## References

[ref1] Macarron R.; Banks M. N.; Bojanic D.; Burns D. J.; Cirovic D. A.; Garyantes T.; Green D. V. S.; Hertzberg R. P.; Janzen W. P.; Paslay J. W.; Schopfer U.; Sittampalam G. S. Impact of high-throughput screening in biomedical research. Nat. Rev. Drug Discovery 2011, 10 (3), 188–195. 10.1038/nrd3368. 21358738

[ref2] Wildey M. J.; Haunso A.; Tudor M.; Webb M.; Connick J. H. High-throughput screening. Annu. Rep. Med. Chem. 2017, 50, 149–195. 10.1016/bs.armc.2017.08.004.

[ref3] Eder J.; Sedrani R.; Wiesmann C. The discovery of first-in-class drugs: Origins and evolution. Nat. Rev. Drug Discovery 2014, 13 (8), 577–587. 10.1038/nrd4336. 25033734

[ref4] Ortwine D. F.; Aliagas I. Physicochemical and dmpk in silico models: Facilitating their use by medicinal chemists. Mol. Pharmaceutics. 2013, 10 (4), 1153–1161. 10.1021/mp3006193. 23402361

[ref5] Mehta D. C.; Short J. L.; Hilmer S. N.; Nicolazzo J. A. Drug access to the central nervous system in Alzheimer’s disease: Preclinical and clinical insights. Pharm. Res. 2015, 32, 819–839. 10.1007/s11095-014-1522-0. 25319097

[ref6] Gribkoff V. K.; Kaczmarek L. K. The need for new approaches in CNS drug discovery: Why drugs have failed, and what can be done to improve outcomes. Neuropharmacology. 2017, 120, 11–19. 10.1016/j.neuropharm.2016.03.021. 26979921 PMC5820030

[ref7] Pangalos M. N.; Schechter L. E.; Hurko O. Drug development for CNS disorders: Strategies for balancing risk and reducing attrition. Nat. Rev. Drug Discovery 2007, 6 (7), 521–532. 10.1038/nrd2094. 17599084

[ref8] Danon J. J.; Reekie T. A.; Kassiou M. Challenges and opportunities in central nervous system drug discovery. Trends Chem. 2019, 1 (6), 612–624. 10.1016/j.trechm.2019.04.009.

[ref9] Pardridge W. M. Drug transport across the blood-brain barrier. J. Cereb. Blood Flow Metab. 2012, 32 (11), 1959–1972. 10.1038/jcbfm.2012.126. 22929442 PMC3494002

[ref10] Pandit R.; Chen L.; Götz J. The blood-brain barrier: Physiology and strategies for drug delivery. Adv. Drug Delivery Rev. 2020, 165, 1–14. 10.1016/j.addr.2019.11.009. 31790711

[ref11] Bender A.; Scheiber J.; Glick M.; Davies J. W.; Azzaoui K.; Hamon J.; Urban L.; Whitebread S.; Jenkins J. L. Analysis of pharmacology data and the prediction of adverse drug reactions and off-target effects from chemical structure. ChemMedChem. 2007, 2 (6), 861–873. 10.1002/cmdc.200700026. 17477341

[ref12] Kramer J. A.; Sagartz J. E.; Morris D. L. The application of discovery toxicology and pathology towards the design of safer pharmaceutical lead candidates. Nat. Rev. Drug Discovery 2007, 6 (8), 636–649. 10.1038/nrd2378. 17643090

[ref13] Seal S.; Carreras-Puigvert J.; Trapotsi M.-A.; Yang H.; Spjuth O.; Bender A. Integrating cell morphology with gene expression and chemical structure to aid mitochondrial toxicity detection. Comm. Biol. 2022, 5 (1), 858 10.1038/s42003-022-03763-5. PMC9399120 35999457

[ref14] Seal S.; Yang H.; Vollmers L.; Bender A. Comparison of cellular morphological descriptors and molecular fingerprints for the prediction of cytotoxicity-and proliferation-related assays. Chem. Res. Toxicol. 2021, 34 (2), 422–437. 10.1021/acs.chemrestox.0c00303. 33522793

[ref15] Fusco G.; Chen S. W.; Williamson P. T. F.; Cascella R.; Perni M.; Jarvis J. A.; Cecchi C.; Vendruscolo M.; Chiti F.; Cremades N.; Ying L.; Dobson C. M.; De Simone A. Structural basis of membrane disruption and cellular toxicity by α-synuclein oligomers. Science. 2017, 358 (6369), 1440–1443. 10.1126/science.aan6160. 29242346

[ref16] Choi M. L.; Chappard A.; Singh B. P.; Maclachlan C.; Rodrigues M.; Fedotova E. I.; Berezhnov A. V.; De S.; Peddie C. J.; Athauda D.; Virdi G. S.; Zhang W.; Evans J. R.; Wernick A. I.; Zanjani Z. S.; Angelova P. R.; Esteras N.; Vinokurov A. Y.; Morris K.; Jeacock K.; Tosatto L.; Little D.; Gissen P.; Clarke D. J.; Kunath T.; Collinson L.; Klenerman D.; Abramov A. Y.; Horrocks M. H.; Gandhi S. Pathological structural conversion of α-synuclein at the mitochondria induces neuronal toxicity. Nat. Neurosci. 2022, 25 (9), 1134–1148. 10.1038/s41593-022-01140-3. 36042314 PMC9448679

[ref17] Gaspar R.; Meisl G.; Buell A. K.; Young L.; Kaminski C. F.; Knowles T. P.; Sparr E.; Linse S. Secondary nucleation of monomers on fibril surface dominates α-synuclein aggregation and provides autocatalytic amyloid amplification. Q. Rev. Bioph. 2017, 50, e6 10.1017/S0033583516000172. 29233218

[ref18] Chia S.; Faidon Brotzakis Z.; Horne R. I.; Possenti A.; Mannini B.; Cataldi R.; Nowinska M.; Staats R.; Linse S.; Knowles T. P. J.; Habchi J.; Vendruscolo M. Structure-based discovery of small-molecule inhibitors of the autocatalytic proliferation of α-Synuclein aggregates. Mol. Pharmaceutics. 2023, 20 (1), 183–193. 10.1021/acs.molpharmaceut.2c00548. PMC9811465 36374974

[ref19] Staats R.; Michaels T. C.; Flagmeier P.; Chia S.; Horne R. I.; Habchi J.; Linse S.; Knowles T. P.; Dobson C. M.; Vendruscolo M. Screening of small molecules using the inhibition of oligomer formation in α-synuclein aggregation as a selection parameter. Comm. Chem. 2020, 3 (1), 191 10.1038/s42004-020-00412-y. PMC9814678 36703335

[ref20] Horne R. I.; Murtada M. H.; Huo D.; Brotzakis Z. F.; Gregory R. C.; Possenti A.; Chia S.; Vendruscolo M. Exploration and exploitation approaches based on generative machine learning to identify potent small molecule inhibitors of α-synuclein secondary nucleation. J. Chem. Theory Comput 2023, 19, 4701 10.1021/acs.jctc.2c01303. 36939645 PMC10373478

[ref21] Horne R. I.; Andrzejewska E.; Alam P.; Brotzakis Z. F.; Srivastava A.; Aubert A.; Nowinska M.; Gregory R. C.; Staats R.; Possenti A. Discovery of potent inhibitors of α-Synuclein aggregation using structure-based iterative learning. bioRxiv Preprint, 2021. 10.1101/2021.11.10.468009, PMC11062903 38632492

[ref22] Wager T. T.; Hou X.; Verhoest P. R.; Villalobos A. Central nervous system multiparameter optimization desirability: Application in drug discovery. ACS Chem. Neurosci. 2016, 7 (6), 767–775. 10.1021/acschemneuro.6b00029. 26991242

[ref47] Liu A.; Seal S.; Yang H.; Bender A. Using Chemical and Biological Data to Predict Drug Toxicity. SLAS Discovery 2023, 28 (3), 53–64. 36639032 10.1016/j.slasd.2022.12.003

[ref23] Kearnes S.; McCloskey K.; Berndl M.; Pande V.; Riley P. Molecular graph convolutions: Moving beyond fingerprints. J. Comput. Aided Mol. Des. 2016, 30, 595–608. 10.1007/s10822-016-9938-8. 27558503 PMC5028207

[ref24] Bray M.-A.; Singh S.; Han H.; Davis C. T.; Borgeson B.; Hartland C.; Kost-Alimova M.; Gustafsdottir S. M.; Gibson C. C.; Carpenter A. E. Cell painting, a high-content image-based assay for morphological profiling using multiplexed fluorescent dyes. Nat. Protoc. 2016, 11 (9), 1757–1774. 10.1038/nprot.2016.105. 27560178 PMC5223290

[ref48] Seal S.; Yang H.; Trapotsi M. A.; Singh S.; Carreras-Puigvert J.; Spjuth O.; Bender A. Merging Bioactivity Predictions from Cell Morphology and Chemical Fingerprint Models Using Similarity to Training Data. J. Chemiinform 2022, 15 (1), 56. 10.1186/s13321-023-00723-x PMC10236827 37268960

[ref25] Zhou Z.; Kearnes S.; Li L.; Zare R. N.; Riley P. Optimization of molecules via deep reinforcement learning. Sci. Rep. 2019, 9 (1), 10752 10.1038/s41598-019-47148-x. 31341196 PMC6656766

[ref26] Maziarka Ł.; Pocha A.; Kaczmarczyk J.; Rataj K.; Danel T.; Warchoł M. Mol-cyclegan: A generative model for molecular optimization. J. Cheminform. 2020, 12 (1), 1–18. 10.1186/s13321-019-0404-1. 33431006 PMC6950853

[ref27] Brown A. S.; Patel C. J. A standard database for drug repositioning. Sci. Data. 2017, 4 (1), 1–7. 10.1038/sdata.2017.29. PMC5349249 28291243

[ref28] Corsello S. M; Bittker J. A; Liu Z.; Gould J.; McCarren P.; Hirschman J. E; Johnston S. E; Vrcic A.; Wong B.; Khan M.; Asiedu J.; Narayan R.; Mader C. C; Subramanian A.; Golub T. R The drug repurposing hub: A next-generation drug library and information resource. Nat. Med. 2017, 23 (4), 405–408. 10.1038/nm.4306. 28388612 PMC5568558

[ref29] Wagner J.; Ryazanov S.; Leonov A.; Levin J.; Shi S.; Schmidt F.; Prix C.; Pan-Montojo F.; Bertsch U.; Mitteregger-Kretzschmar G.; Geissen M.; Eiden M.; Leidel F.; Hirschberger T.; Deeg A. A.; Krauth J. J.; Zinth W.; Tavan P.; Pilger J.; Zweckstetter M.; Frank T.; Bähr M.; Weishaupt J. H.; Uhr M.; Urlaub H.; Teichmann U.; Samwer M.; Bötzel K.; Groschup M.; Kretzschmar H.; Griesinger C.; Giese A. Anle138b: A novel oligomer modulator for disease-modifying therapy of neurodegenerative diseases such as prion and Parkinson’s disease. Acta Neuropathol. 2013, 125, 795–813. 10.1007/s00401-013-1114-9. 23604588 PMC3661926

[ref30] Trott O.; Olson A. J. Autodock vina: Improving the speed and accuracy of docking with a new scoring function, efficient optimization, and multithreading. J. Comput. Chem. 2010, 31 (2), 455–461. 10.1002/jcc.21334. 19499576 PMC3041641

[ref31] Li B.; Ge P.; Murray K. A.; Sheth P.; Zhang M.; Nair G.; Sawaya M. R.; Shin W. S.; Boyer D. R.; Ye S.; Eisenberg D. S.; Zhou Z. H.; Jiang L. Cryo-EM of full-length α-synuclein reveals fibril polymorphs with a common structural kernel. Nat. Commun. 2018, 9 (1), 3609 10.1038/s41467-018-05971-2. 30190461 PMC6127345

[ref32] Yang Y.; Shi Y.; Schweighauser M.; Zhang X.; Kotecha A.; Murzin A. G.; Garringer H. J.; Cullinane P. W.; Saito Y.; Foroud T.; Warner T. T.; Hasegawa K.; Vidal R.; Murayama S.; Revesz T.; Ghetti B.; Hasegawa M.; Lashley T.; Scheres S. H. W.; Goedert M. Structures of α-synuclein filaments from human brains with Lewy pathology. Nature. 2022, 610 (7933), 791–795. 10.1038/s41586-022-05319-3. 36108674 PMC7613749

[ref33] Kurnik M.; Sahin C.; Andersen C. B.; Lorenzen N.; Giehm L.; Mohammad-Beigi H.; Jessen C. M.; Pedersen J. S.; Christiansen G.; Petersen S. V.; Staal R.; Krishnamurthy G.; Pitts K.; Reinhart P. H.; Mulder F. A.A.; Mente S.; Hirst W. D.; Otzen D. E. Potent α-synuclein aggregation inhibitors, identified by high-throughput screening, mainly target the monomeric state. Cell Chem. Biol. 2018, 25 (11), 1389–1402. 10.1016/j.chembiol.2018.08.005. 30197194

[ref34] Hie B.; Bryson B. D.; Berger B. Leveraging uncertainty in machine learning accelerates biological discovery and design. Cell Syst. 2020, 11 (5), 461–477. 10.1016/j.cels.2020.09.007. 33065027

[ref35] Irwin J. J.; Shoichet B. K. Zinc- a free database of commercially available compounds for virtual screening. J. Chem. Inf. Model. 2005, 45 (1), 177–182. 10.1021/ci049714+. 15667143 PMC1360656

[ref36] Irwin J. J.; Gaskins G.; Sterling T.; Mysinger M. M.; Keiser M. J. Predicted biological activity of purchasable chemical space. J. Chem. Inf. Model. 2018, 58 (1), 148–164. 10.1021/acs.jcim.7b00316. 29193970 PMC5780839

[ref37] Huang T.; Mi H.; Lin C.-Y.; Zhao L.; Zhong L. L.; Liu F.-B.; Zhang G.; Lu A.-P.; Bian Z.-X. MOST: Most-similar ligand based approach to target prediction. BMC Bioinformatics. 2017, 18, 1–11. 10.1186/s12859-017-1586-z. 28284192 PMC5346209

[ref38] Muchmore S. W.; Debe D. A.; Metz J. T.; Brown S. P.; Martin Y. C.; Hajduk P. J. Application of belief theory to similarity data fusion for use in analog searching and lead hopping. J. Chem. Inf. Model. 2008, 48 (5), 941–948. 10.1021/ci7004498. 18416545

[ref39] Wang L.; Ma C.; Wipf P.; Liu H.; Su W.; Xie X.-Q. Targethunter: An in silico target identification tool for predicting therapeutic potential of small organic molecules based on chemogenomic database. AAPS J. 2013, 15, 395–406. 10.1208/s12248-012-9449-z. 23292636 PMC3675739

[ref40] Chia S.; Habchi J.; Michaels T. C.; Cohen S. I.; Linse S.; Dobson C. M.; Knowles T. P.; Vendruscolo M. SAR by kinetics for drug discovery in protein misfolding diseases. Proc. Natl. Acad. Sci. U.S.A. 2018, 115 (41), 10245–10250. 10.1073/pnas.1807884115. 30257937 PMC6187117

[ref41] Moret M.; Friedrich L.; Grisoni F.; Merk D.; Schneider G. Generative molecular design in low data regimes. Nat. Mach. Intell. 2020, 2 (3), 171–180. 10.1038/s42256-020-0160-y.

[ref42] McKinney W. Data Structures for Statistical Computing in Python. In Proceedings of the 9th Python in Science Conference, Austin, TX, 2010; pp 51–56.

[ref43] Waskom M. L. Seaborn: Statistical data visualization. J. Open Source Softw. 2021, 6 (60), 3021 10.21105/joss.03021.

[ref44] Hunter J. D. Matplotlib: A 2d graphics environment. Comput. Sci. Eng. 2007, 9 (03), 90–95. 10.1109/MCSE.2007.55.

[ref45] Harris C. R.; Millman K. J.; van der Walt S. J.; Gommers R.; Virtanen P.; Cournapeau D.; Wieser E.; Taylor J.; Berg S.; Smith N. J.; Kern R.; Picus M.; Hoyer S.; van Kerkwijk M. H.; Brett M.; Haldane A.; del Rio J. F.; Wiebe M.; Peterson P.; Gerard-Marchant P.; Sheppard K.; Reddy T.; Weckesser W.; Abbasi H.; Gohlke C.; Oliphant T. E. Array programming with numpy. Nature. 2020, 585 (7825), 357–362. 10.1038/s41586-020-2649-2. 32939066 PMC7759461

[ref46] Virtanen P.; Gommers R.; Oliphant T. E.; Haberland M.; Reddy T.; Cournapeau D.; Burovski E.; Peterson P.; Weckesser W.; Bright J.; et al. Scipy 1.0: Fundamental algorithms for scientific computing in python. Nat. Methods. 2020, 17 (3), 261–272. 10.1038/s41592-019-0686-2. 32015543 PMC7056644

