# Supplementary material for: Using Generative Modeling to Endow with Potency Initially Inert Compounds with Good Bioavailability and Low Toxicity
Source: J Chem Inf Model. 2024 Jan 23;64(3):590–6. doi: 10.1021/acs.jcim.3c01777 (PMC10865343; doi:10.1021/acs.jcim.3c01777)
Supplement: Supplementary file 1 — ci3c01777_si_001.pdf [file ci3c01777_si_001.pdf]

SUPPORTING INFORMATION

Using machine learning to endow with potency initially inert compounds with good bioavailability and low toxicity

Robert I. Horne[1*], Jared Wilson-Godber[1*], Alicia González Díaz[1], Z. Faidon Brotzakis[1], Srijit Seal[1,2], Rebecca C. Gregory[1], Andrea Possenti[1], Sean Chia[1,3], Michele Vendruscolo[1+]

[1]Centre for Misfolding Diseases, Department of Chemistry, University of Cambridge, Cambridge CB2 1EW, UK

[2]Imaging Platform, Broad Institute of MIT and Harvard, Cambridge, Massachusetts 02142, United States

*[3]Bioprocessing Technology Institute, Agency of Science, Technology and Research (A*STAR), Singapore*

**Authors contributed equally to this manuscript*

[+]mv245@cam.ac.uk

Keywords

Drug discovery; Machine learning; Screening; Drug metabolism and pharmacokinetics; Toxicity prediction; Structural elaboration

SUPPORTING INFORMATION

Experimental Methods

Compounds and chemicals

Compounds were purchased from MolPort (Riga, Latvia) or Mcule, and prepared in DMSO to a stock of 5 mM. All chemicals used were purchased at the highest purity available.

Recombinant αS expression

Recombinant αS was purified as described previously[1-3]. The plasmid pT7-7 encoding for human αS was transformed into BL21-competent cells. Following transformation, competent cells were grown in LB in the presence of ampicillin (100 µg/mL). Cells were induced with IPTG and grown overnight at 37 ^{o}C and harvested by centrifugation in a Beckman Avanti J25 centrifuge with a JA-20 rotor at 5000 rpm (Beckman Coulter, Fullerton, CA). The cell pellet was resuspended in 10 mM Tris, pH 8.0, 1 mM EDTA, 1 mM PMSF and lysed by multiple freeze–thaw cycles and sonication. The cell suspension was boiled for 20 min and centrifuged at 13,500 rpm with a JA-20 rotor (Beckman Coulter). Streptomycin sulfate was added to the supernatant to a final concentration of 10 mg/mL and the mixture was stirred for 15 min at 4 ^{o}C. After centrifugation at 13,500 rpm, the supernatant was taken with an addition of 0.36 g/mL ammonium sulfate. The solution was stirred for 30 min at 4 ^{o}C and centrifuged again at 13,500 rpm. The pellet was resuspended in 25 mM Tris, pH 7.7, and ion-exchange chromatography was performed using a HQ/M-column of buffer A (25 mM Tris, pH 7.7) and buffer B (25 mM Tris, pH 7.7, 600 mM NaCl). The fractions containing αS (≈ 300 µM) were dialysed overnight against the appropriate buffer. The protein concentration was determined spectrophotometrically using ε280 = 5600 M^{-1} cm^{-1}.

Seed fibril preparation

αS fibril seeds were produced as described previously[1, 2]. Samples of αS (700 µM) were incubated in 20 mM phosphate buffer (pH 6.5) for 72 h at 40 °C and stirred at 1,500 rpm with a Teflon bar on an RCT Basic Heat Plate (IKA, Staufen, Germany). Fibrils were then diluted to 200 µM, aliquoted and flash frozen in liquid N_2, and finally stored at -80 ^{o}C. For the use of kinetic experiments, the 200 µM fibril stock was thawed, and

sonicated for 15 s using a tip sonicator (Bandelin, Sonopuls HD 2070, Berlin, Germany), using 10% maximum power and a 50% cycle.

Measurement of aggregation kinetics

αS was injected into a Superdex 75 10/300 GL column (GE Healthcare) at a flow rate of 0.5 mL/min and eluted in 20 mM sodium phosphate buffer (pH 4.8) supplemented with 1 mM EDTA. The obtained monomer was diluted in buffer to a desired concentration and supplemented with 50 µM ThT and preformed αS fibril seeds. The molecules (or DMSO alone) were then added at the desired concentration to a final DMSO concentration of 1% (v/v). Samples were prepared in low-binding Eppendorf tubes, and then pipetted into a 96-well half-area, black/clear flat bottom polystyrene NBS microplate (Corning 3881), 150 µL per well. The assay was then initiated by placing the microplate at 37 ^{o}C under quiescent conditions in a plate reader (FLUOstar Omega, BMG Labtech, Aylesbury, UK). The ThT fluorescence was measured through the bottom of the plate with a 440 nm excitation filter and a 480 nm emission filter. After centrifugation at 5000 rpm to remove aggregates the monomer concentration was measured via the Pierce™ BCA Protein Assay Kit according to the manufacturer's protocol.

Toxicity measurement

Human neuroblastoma cells (SH-SY5Y) were cultured in DMEM/F-12 GlutaMAX™ (#10565018, Gibco) supplemented with 10% heat inactivated foetal bovine serum (hiFBS) (#10082147, Gibco). Cells were kept at 37°C, 5% CO2 and 95% relative humidity, unless otherwise stated. For each experiment, cells were plated in tissue culture-treated, flat bottom, white polystyrene plates (#CLS3917, Corning) at a final density of 10k or 20k cells/well. Cells were incubated for 24h prior to treatment to ensure attachment. On the day of the treatment, drugs were sonicated for 10 minutes and filtered using a 0.02 µm Whatman Anotop filter. 100X stocks from each molecule were prepared in a sterile mirror plate (#CLS3997, Corning) by performing log-serial dilutions in DMSO. Intermediate drug stocks were diluted in serum free DMEM/F-12 GlutaMAX™ to a final 10X concentration. Mirror plates were incubated for 10 minutes at 550 rpm. 30 minutes prior to the addition of the drugs, culture medium was replaced with serum free DMEM/F-12 GlutaMAX™. 90 µl of medium was added per well. 10µl of each drug

intermediate stock was added to the corresponding wells, keeping a final % v/v of DMSO at 1%. Cells were incubated in the presence of the drugs for 24h. CellTiterGlo (CTG) reagent was prepared as detailed by the manufacturer (#G7570, Promega). Plates were equilibrated at room temperature for approximately 30 minutes. 100µl of CTG solution was added per well. Cells were incubated for 2 minutes under shaking conditions to aid lysis. Subsequently, plates were kept at room temperature for 10 minutes prior to luminescence recording using a plate reader (CLARIOstar, BMG Labtech).

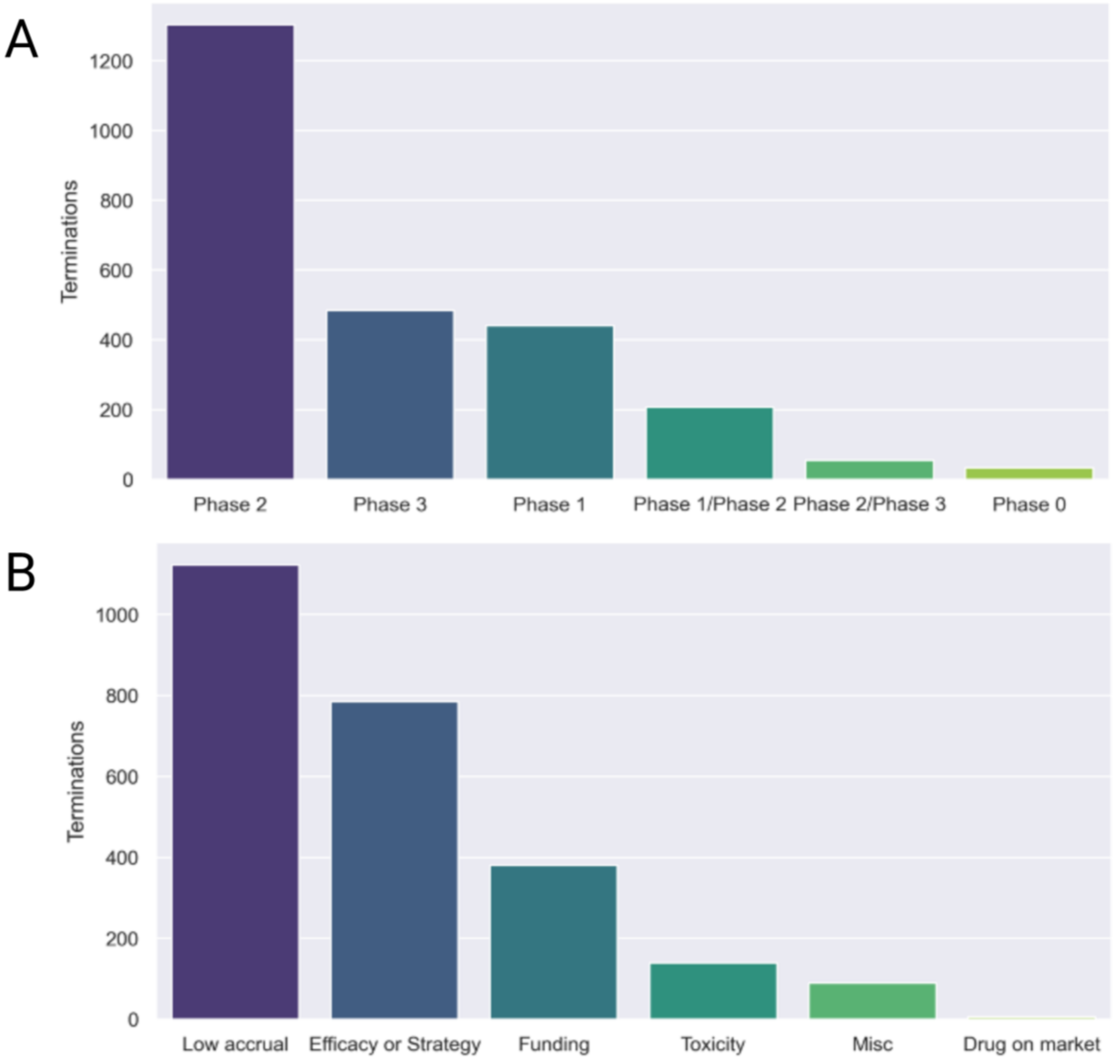

Figure S1. Null library from a repositioning database. Summary of drug trial terminations by (**A**) stage of progression and by (**B**) cause of termination. Molecules reaching at least phase 2 without failure due to toxicity were considered viable candidates for repurposing. 'Low accrual' meant the trial was terminated due to poor recruitment for the trial, 'efficacy or strategy' meant there was a decision by the trial runners to terminate due to failure to meet end points or altered priorities within the company, while 'drug on market' meant an effective drug had become available for the target of the trial during the trial's running.

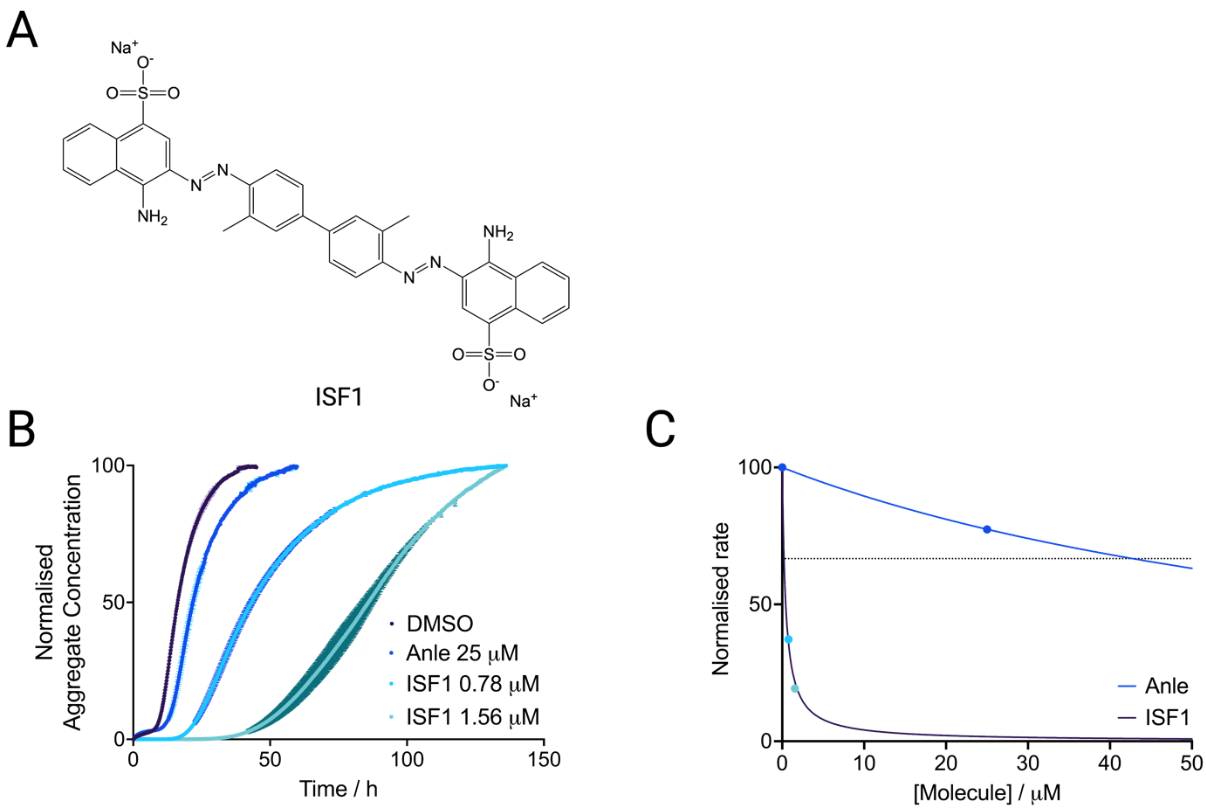

Figure S2. Most potent compound obtained using the second strategy of direct in silico toxicity/potency library screening. (A) Structure of ISF1. **(B)** Kinetic traces of a 10 µM solution of αS with 25 nM seeds at pH 4.8, 37°C in the presence of ISF1 at the concentration indicated (different colours) or 1% DMSO (purple). Anle-138b is shown as a positive control. **(C)** Approximate rate of reaction (taken as $1/t_{1/2}$, normalised between 0 and 100) in the presence of 2 different molecules, Anle-138b (blue) and ISF1 (purple). The data point colours match those in **B**. The KIC_{50} of ISF1 (0.22 µM) is indicated by the intersection of the fit and the horizontal dotted line. Anle-138b has an extrapolated KIC_{50} of 42.86 µM based on the sample tested here.

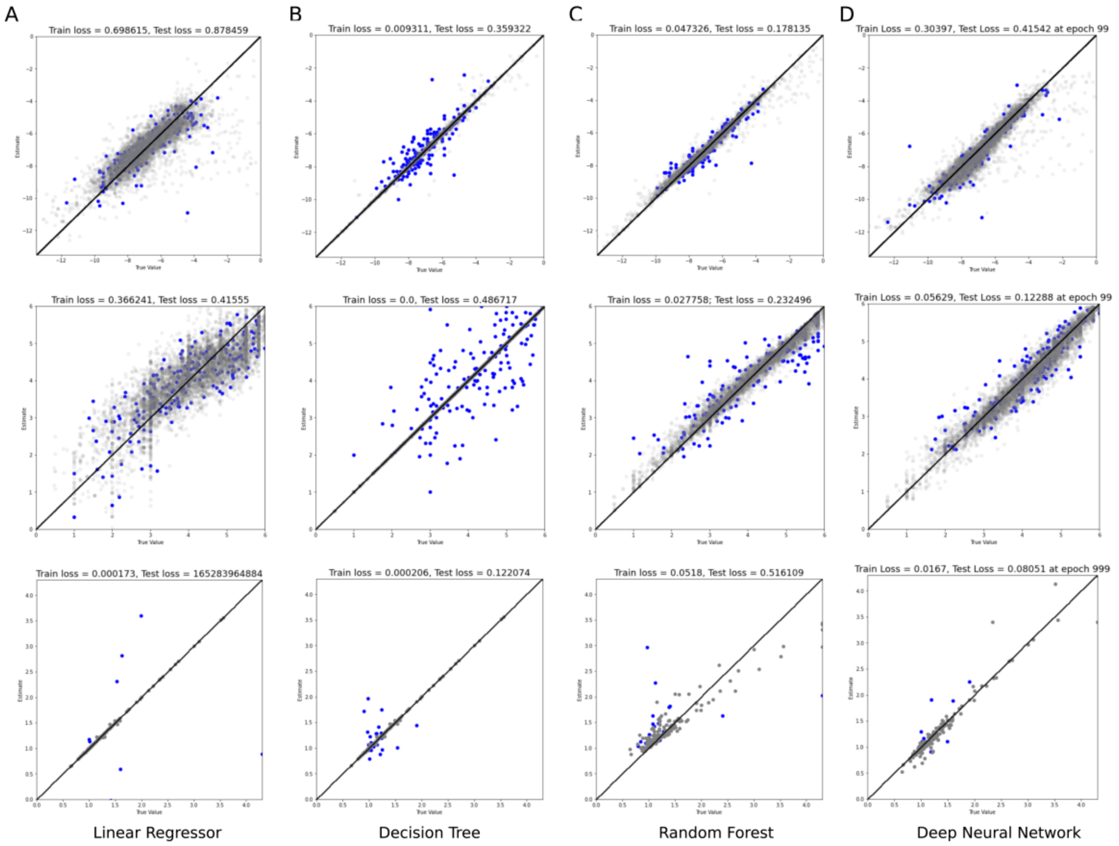

Figure S3: Comparison of different QSAR models for the prediction of the AutoDock Vina score (top), CNS MPO score (middle) and half-time of aggregation (bottom). (A) Linear regressor **(B)** Decision tree regressor **(C)** Random Forest regressor **(D)** Deep neural network. Example graphs are shown for training runs. Grey points represent molecules in the training set for each model, blue points represent molecules in the testing set, with true values on the x-axis and model estimates on the y-axis. The cross validation average MSE for training and testing is indicated above each graph.

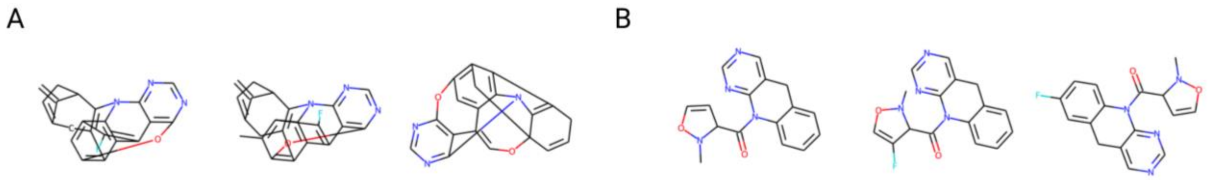

Figure S4: (A) Examples of generated molecules before introduction of synthetic accessibility parameter optimisation. **(B)** Examples of generated molecules after introduction of synthetic accessibility parameter optimisation.

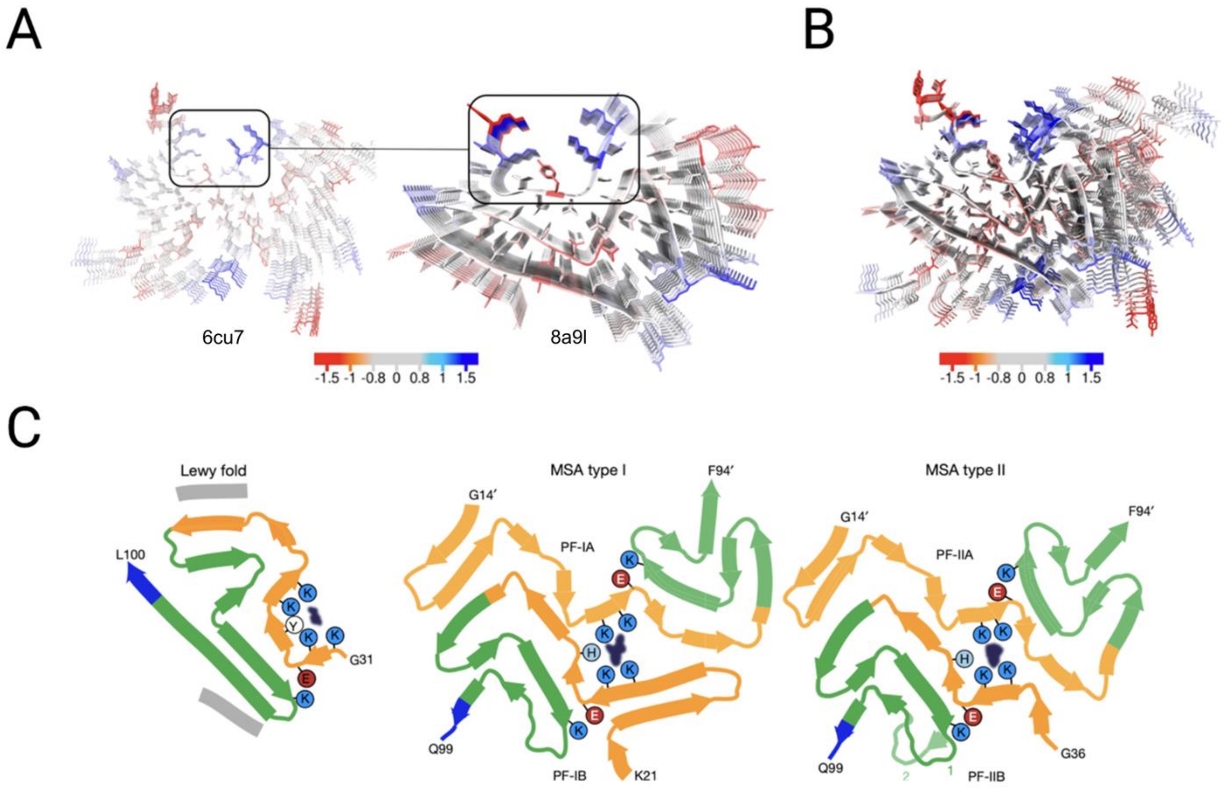

Figure S5: (A) Comparison of the cryo-EM structures of the 6cu7 (recombinant, initially targeted) and 8a9l (brain derived) with the homologous binding site indicated. **(B)** Structural overlap of the 6cu7 and 8a9l fibril structures, with the binding site in 6cu7 aligned with the similar binding site in 8a9l at the top of the diagram. The structures are coloured according to the CamSol residue solubility score[4]. **(C)** Folds of the prevalent fibril polymorph in diseased brain material identified via cryo-EM in Parkinson's disease and dementia with Lewy bodies (8a9l), MSA type I and MSA type II. A common motif of 4 lysines enclosing an aromatic side chain (tyrosine in the Lewy fold and histidine in the MSA fold and 6cu7) is observed in the polymorphs, with unidentified electron density in the pocket in each case (adapted from reference [5]).

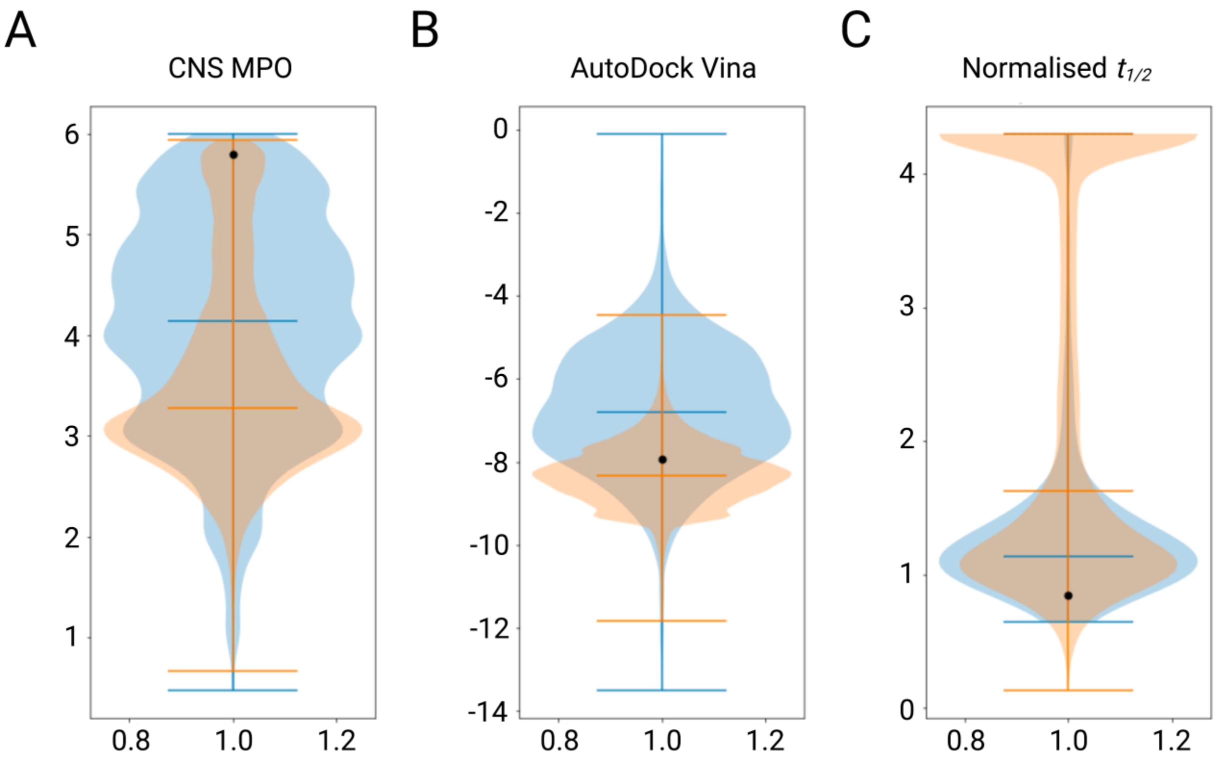

Figure S6. Comparison of the training set distribution (blue) and the predicted test set distribution (orange) for the 3 different parameters that were optimised in parallel. (**A**) CNS MPO values for the Cayman dataset consist of the sum of 6 parameters scored between 0 and 1, with values closer to 1 being more desirable for penetration of the blood brain barrier. The inert starting molecule is shown as a black point and model was trained for 100 epochs before generating the set of molecules shown here (orange). (**B**) AutoDock Vina docking scores for the Cayman dataset (middle) are measured in kcal mol^{-1} and reflect the predicted binding to a binding pocket found in the 6cu7 (recombinant fibril) and 8a9l (brain derived) fibril structures. The inert starting molecule is shown as a black point and model was trained for 100 epochs before generating the set of molecules shown here (orange). (**C**) Normalised half times of aggregation for the aggregation dataset (right) correspond to the metric of potency in the αS secondary nucleation assay. A value of 1 implies no effect, while the most efficacious hits increased the half time by 4-5 times that of the negative control. The inert starting molecule is shown as a black point and model was trained for 1000 epochs before generating the set of molecules shown here (orange).

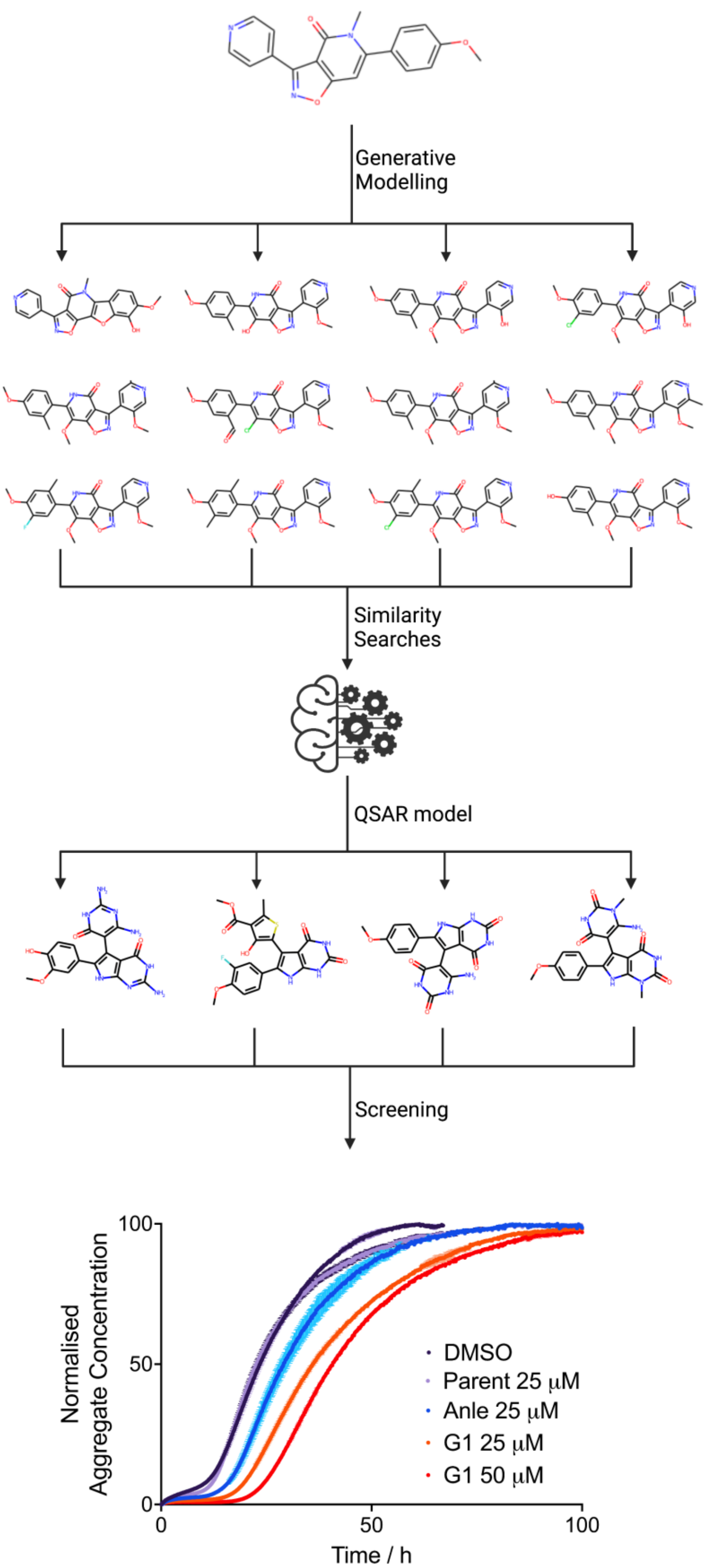

Figure S7. Summary of the route to the aggregation inhibitors. Initial generative modelling via MolDQN provided new molecules based on the starting structure that were predicted to have higher potency, AutoDock Vina score and good CNS MPO.

SUPPORTING INFORMATION

Similarity searches for the best predicted molecules from this set on the ZINC15 database produced a new dataset, which was screened via a previously developed QSAR model to identify potential hits. Of the 7 molecules purchased and tested, 4 showed anti-aggregation activity in the αS secondary nucleation assay. The dose-dependent effect of one of these 4 molecules (G1) is shown (kinetic traces of a 10 µM solution of αS with 25 nM seeds at pH 4.8, 37 °C in the presence of molecule or 1% DMSO).

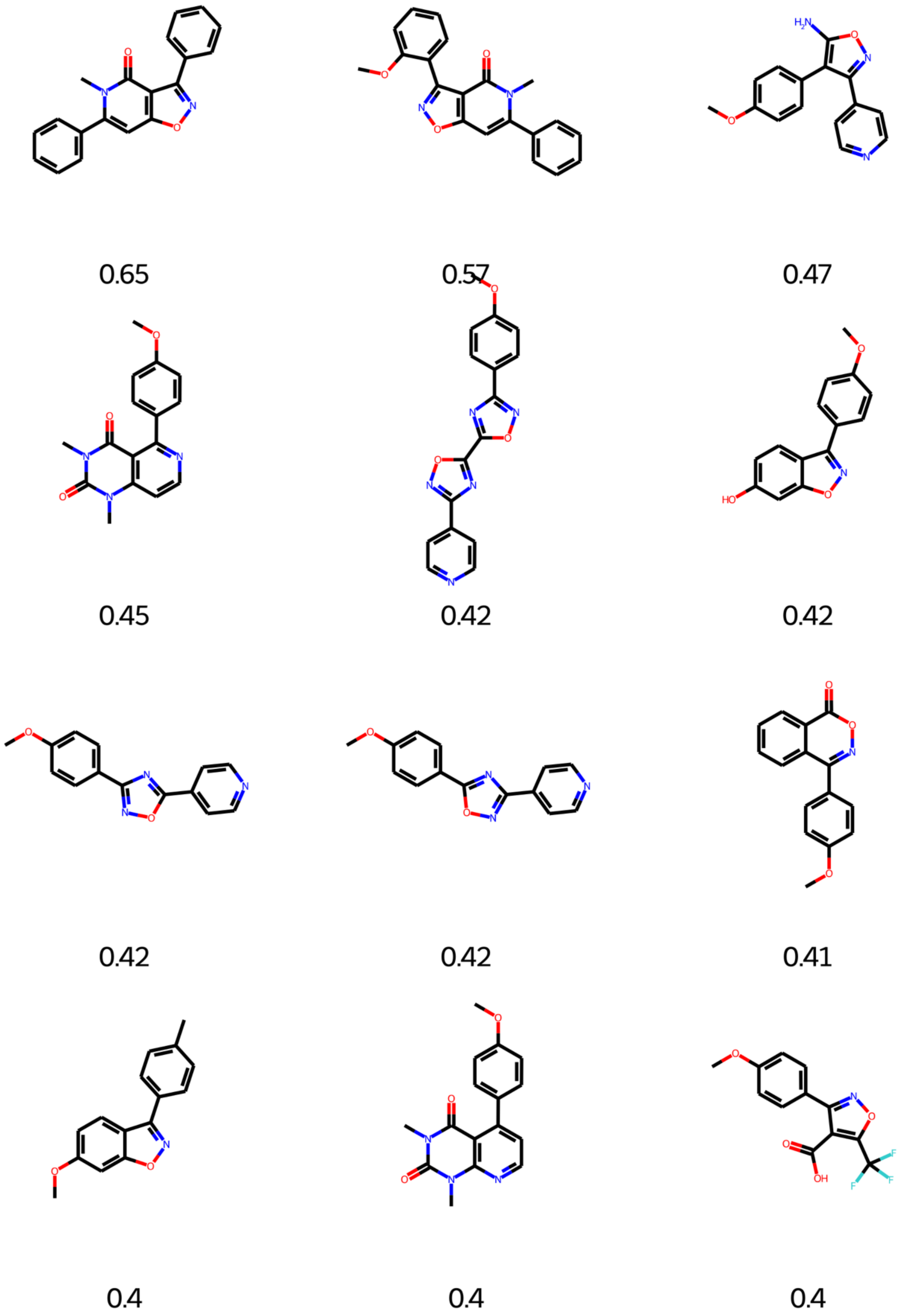

Figure S8: The top 12 most similar structures to the parent molecule within the ZINC dataset according to Tanimoto similarity (nbits=2048, radius=2). Tanimoto similarity values (0-1, 1 being an exact match) to the parent are indicated below each structure.

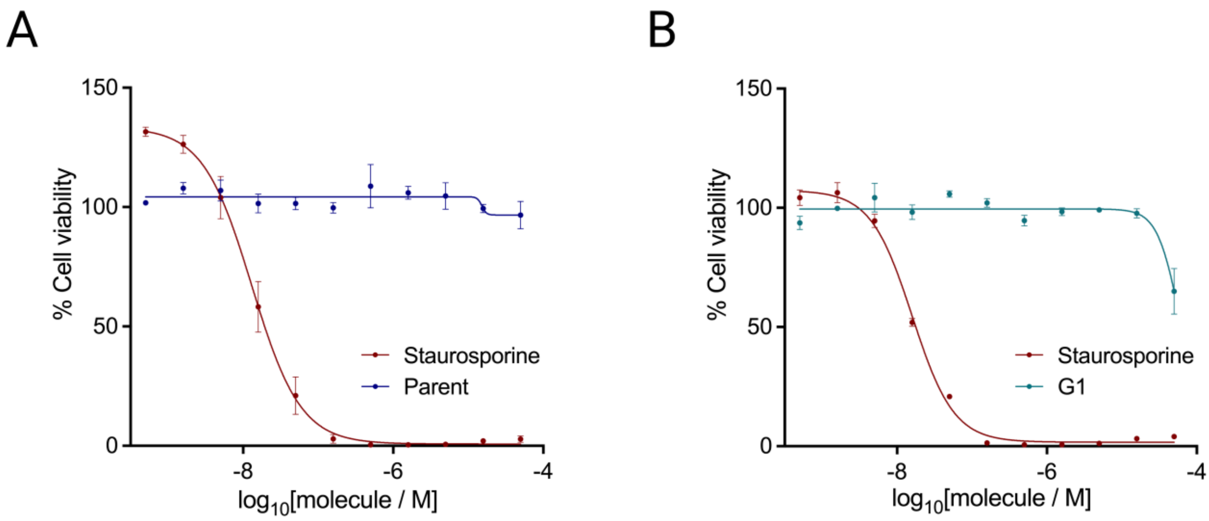

Figure S9. Human neuroblastoma cells (SH-SY5Y) at a final density of 10k cells/well and incubated in the presence of (**A**) the generative modelling parent molecule or (**B**) G1 for 24h, before addition of CellTiterGlo to detect ATP levels as a proxy for cell viability. The concentration range is shown as a log scale, from 500 pM to 50 μM. Staurosporine, which induces apoptosis, is shown as a negative control.

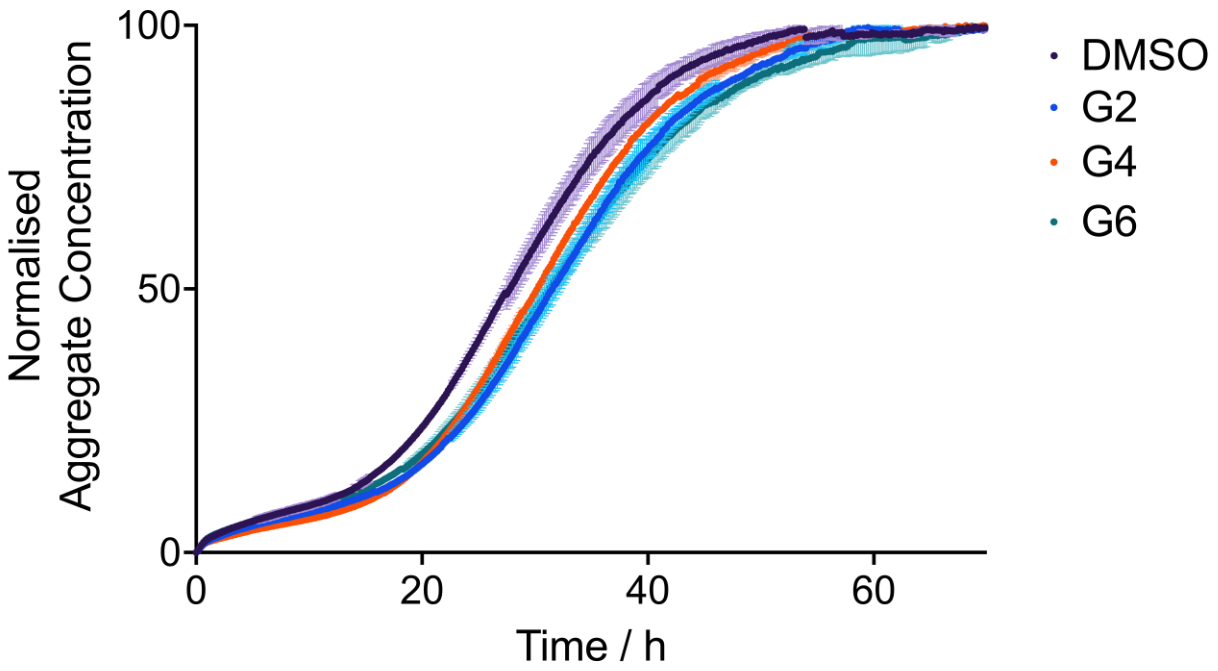

Figure S10. 3 other molecules exhibiting potency from the generative modelling. Kinetic traces of a 10 µM solution of αS with 25 nM seeds at pH 4.8, 37°C in the presence of molecule (different colours) or 1% DMSO (purple). These molecules exhibited milder potency with a 10-15% increase in half time at 25 µM, compared with a 25%-30% increase for Anle-138b at 25 µM.

SUPPORTING INFORMATION

References

(1) Buell, A. K.; Galvagnion, C.; Gaspar, R.; Sparr, E.; Vendruscolo, M.; Knowles, T. P.; Linse, S.; Dobson, C. M. Solution conditions determine the relative importance of nucleation and growth processes in alpha-synuclein aggregation. *Proc Natl Acad Sci U S A* **2014**, *111* (21), 7671-7676.
(2) Flagmeier, P.; Meisl, G.; Vendruscolo, M.; Knowles, T. P.; Dobson, C. M.; Buell, A. K.; Galvagnion, C. Mutations associated with familial Parkinson's disease alter the initiation and amplification steps of alpha-synuclein aggregation. *Proc Natl Acad Sci U S A* **2016**, *113* (37), 10328-10333.
(3) Galvagnion, C.; Buell, A. K.; Meisl, G.; Michaels, T. C.; Vendruscolo, M.; Knowles, T. P.; Dobson, C. M. Lipid vesicles trigger α-synuclein aggregation by stimulating primary nucleation. *Nature chemical biology* **2015**, *11* (3), 229-234.
(4) Sormanni, P.; Aprile, F. A.; Vendruscolo, M. The CamSol method of rational design of protein mutants with enhanced solubility. *J Mol Biol* **2015**, *427* (2), 478-490.
(5) Yang, Y.; Shi, Y.; Schweighauser, M.; Zhang, X.; Kotecha, A.; Murzin, A. G.; Garringer, H. J.; Cullinane, P. W.; Saito, Y.; Foroud, T. Structures of α-synuclein filaments from human brains with Lewy pathology. *Nature* **2022**, *610* (7933), 791-795.
